# Cathepsin S As an Inhibitor of Cardiovascular Inflammation and Calcification in Chronic Kidney Disease

**DOI:** 10.3389/fcvm.2017.00088

**Published:** 2018-01-05

**Authors:** Brena F. Sena, Jose Luiz Figueiredo, Elena Aikawa

**Affiliations:** ^1^Boston University School of Public Health, Boston, MA, United States; ^2^Department of Surgery, Introduction to Clinical and Surgical Techniques Division, Laboratory of Experimental Surgery, Federal University of Pernambuco, Recife, Brazil; ^3^The Center of Excellence in Vascular Biology, Department of Medicine, Brigham and Women’s Hospital, Harvard Medical School, Boston, MA, United States

**Keywords:** cathepsin S, cardiovascular disease, chronic kidney disease, calcification, atherosclerosis, aortic valve

## Abstract

Cardiovascular disease (CVD) is responsible for the majority of deaths in the developed world. Particularly, in patients with chronic kidney disease (CKD), the imbalance of calcium and phosphate may lead to the acceleration of both vascular and valve inflammation and calcification. One in two patients with CKD are reported as dying from cardiovascular causes due to the resulting acceleration in the development of atherosclerosis plaques. In addition, CKD patients on hemodialysis are prone to aortic valve calcification and often need valve replacement before kidney transplantation. The lysosomal proteases, cathepsins, are composed of 11 cysteine members (cathepsin B, C, F, H, K, L, O, S, V, W, and Z), as well as serine proteases cathepsin A and G, which cleave peptide bonds with serine as the amino acid, and aspartyl proteases D and E, which use an activated water molecule bound to aspartate to break peptide substrate. Cysteine proteases, also known as thiol proteases, degrade protein *via* the deprotonation of a thiol and have been found to play a significant role in autoimmune disease, atherosclerosis, aortic valve calcification, cardiac repair, and cardiomyopathy, operating within extracellular spaces. This review sought to evaluate recent findings in this field, highlighting how among cathepsins, the inhibition of cathepsin S in particular, could play a significant role in diminishing the effects of CVD, especially for patients with CKD.

## Introduction

Cathepsins are lysosomal proteases that breakdown peptide bonds linked to specific amino acids. They are known for their key role in protein turnover and significantly contribute to the breakdown of the extracellular matrix (ECM). More importantly, cathepsins play a crucial role in various conditions that involve large biological systems such as autoimmune disease, cardiac repair, cardiomyopathy, heart valve disease, and atherosclerosis.

The cathepsin family, cysteinyl, in specific, is known to date to be composed of 11 members in humans including cathepsins B, C, F, H, K, L, O, S, V, W, and Z. Cathepsin S is thought to be a particularly potent cysteine protease cleaving elastin and generating bioactive elastin peptides, leading to the promotion of cardiovascular inflammation and calcification. Beyond boosting inflammation as a part of its own mechanistic process, cathepsin S is also released by smooth muscle cells and macrophages as a systemic response to inflammation in a continuous recursive feedback loop.

Vascular inflammation, in particular, is a characteristic feature in the initiation and progression of atherosclerotic lesions. Vascular inflammation that is caused by atherosclerosis is described by widespread changes and remodeling of the ECM within the arterial wall, both in the degradation of the matrix and cellular phenotypic changes. Compounded by chronic inflammation and a consistent imbalance in calcium phosphate serum levels, patients with chronic kidney disease (CKD) face a deleterious feedback loop. This harmful combination further accelerates and exacerbates the evolution of atherosclerosis in patients with CKD. Studies on atherosclerosis-associated inflammatory cytokines have also shown that cathepsin expression and activity are amplified within vascular cells (1).

Also affecting patients with CKD, aortic valve calcification is highlighted as a major clinical problem in this population, where calcification can lead to the progression of aortic valve stenosis. Patients with CKD on hemodialysis are prone to developing aortic valve calcification and likely to need valve replacement before undergoing kidney transplantation. Of further importance is the fact that no current treatment options are available for patients with aortic valve stenosis besides valve replacement. Further, recent clinical trials have shown no benefit to statin therapy in halting the progression of aortic calcification and stenosis (2), leaving health professionals and patients with little to no options.

Within this context, one in two patients with CKD are reported as dying from increased cardiovascular burden particularly resulting from an acceleration in the development of calcification and atherosclerotic plaque (3). Hence, the acceleration in the development of calcification and atherosclerosis in patients with CKD is a leading factor in the simultaneously higher cardiovascular risk, the likelihood of developing cardiovascular disease (CVD), and ultimately death from cardiovascular causes. Thus, interventions that could support in halting or reducing calcification and atherosclerosis, especially for patients with CKD is of desperate need.

The inhibition of cathepsin S has been highlighted as a promising intervention in reducing plaque development and diminishing the effects of CVD, especially for patients with CKD (4). This is especially critical considering the lack of treatment options for patients with aortic valve calcification that risk progression to stenosis and the need for valve replacement. Cathepsin S inhibition has been shown in evaluations of various cathepsins and inhibitors as a promising target. Through this review, we hope to better understand the role of cathepsin S in CVD inflammation and calcification in CKD. More specifically, we will analyze inquiries into cathepsin S mechanism of action, its role in various processes of inflammation, calcification, and renal disease, and identify cathepsin S inhibitors that have been gaged as potential and promising treatment targets.

## Materials and Methods

A review was conducted through a search of the PubMed, Medline, the Cochrane Library, and Google databases. The following keywords were used: “cathepsin S,” “cardiovascular disease,” “chronic renal disease,” “cathepsin S inhibitor,” “renal disease,” “calcification,” and “atherosclerosis.” The keywords were combined to reach the relevant results.

A review of the titles and abstracts was undertaken to find those that matched the keywords of the search. Following the review of titles and abstracts, the authors identified six categories of interest and organized the articles based on this classification: “treatment by cathepsin S inhibitor,” “cathepsin S and renal disease,” “cathepsin S mechanism,” “cathepsin S and calcification,” “cathepsin S and inflammation,” and “miscellaneous.”

The initial review of the databases yielded a total of 41 articles. Of those 41, 13 articles were categorized under “cathepsin S mechanism,” 12 under “cathepsin S and inflammation,” 7 articles were categorized under “treatment by cathepsin S inhibitor,” 6 were as “cathepsin S and renal disease,” one under the category “cathepsin S and calcification,” and two under “miscellaneous,”

**Table:** 

Categories	Number of articles	Publications
Cathepsin S mechanism	13	Aikawa et al. (3), Wu et al. (5), Chen et al. (6), Moran et al. (7); Pauly et al. (8), Qin et al. (9), Lv et al. (10), Pai et al. (11), Cheng et al. (1), Sasaki et al. (12), Shi et al. (13), Liuzzo et al. (14), and Shi et al. (15)
Cathepsin S and inflammation	12	Lafarge et al. (16), Reiser et al. (17), Naour et al. (18), Stenvinkel et al. (19), Lutgens et al. (20), Rodgers et al. (21), Liu et al. (22), Basalyga et al. (23), Go et al. (24), Sukhova et al. (25), Jobs et al. (26), and Jobs et al. (27)
Treatment by cathepsin S inhibitor	7	Ahmad and Siddiqi (28), Rupanagudi et al. (29), Payne et al. (30), Jadhav et al. (31), Hilpert et al. (32), Figueiredo et al. (4), and Samokhin et al. (33)
Cathepsin S and renal disease	6	Huang et al. (34), Steubl et al. (35), Carlsson et al. (36), Jobs et al. (37), Smith et al. (38), and Inker et al. (39)
Cathepsin S and calcification	1	Kumar et al. (40)
Miscellaneous	2	Hewitt et al. (41), and Nadkarni et al. (42)

## Results

### Proposed Mechanism of Cathepsin S Action

Researchers have sought to understand the role of cathepsin S in CVD and systems by evaluating the molecule’s potential mechanisms of action. One of the systemic mechanisms recently studied is cathepsin S’s role in cardiovascular inflammation and calcification especially for patients faced with CKD.

Aikawa et al. (3) demonstrate through *in vivo* experiments that elastin degradation induced by cathepsin S accelerates arterial and aortic valve calcification in a CKD model. The findings indicate a mechanism of action where cathepsin S contributes to the cleavage of the elastin matrix in atherosclerotic lesions and inflamed valves, and in the disruption of the tissue layer, mesenchymal cells [vascular smooth muscle cells (SMCs) or valvular myofibroblasts] proliferate and calcify. The researchers highlight that CKD patients with systemic mineral imbalance or hyperphosphatemia experience further acceleration of calcification. The investigators conclude that future interventions in calcification-prone individuals should target inflammation and phosphate imbalance to reduce the effects of negative feedback loops related to elastin degradation and calcification, and incorporate the selective inhibition of cathepsin S as a treatment target.

On the other hand, in an arterial medial calcification (AMC), a different type of vascular calcification, uremic mouse-model fed a high-phosphate diet, Pai et al. (11) suggest that even though elastin degradation is a necessary factor in the cascade leading to AMC, it may not be sufficient on its own to induce calcification. The researchers indicate that in their findings although elastin degradation did occur in uremic mice on a normal phosphate diet, they did not develop AMC and thus elastin degradation would not be sufficient to induce medial calcification. The researchers instead indicate that phenotypic changes and loss of vascular SMCs would be both “necessary and sufficient” culprits to induce AMC and not elastin degradation alone.

Nevertheless, Simionescu et al. (43) in a study looking at the role of fibroblasts in medial vascular calcification find that calcified nodules are formed in the presence of elastin degradation products and transforming growth factor (TGF)-β1, especially when used together. In this way, the investigators show that elastin degradation peptides, highly active biologic products known as matrikines, can induce calcification of mesenchymal cells *in vitro*. These findings suggest that elastin degradation could in fact induce calcification.

Additionally, in a murine model with cathepsin S inhibitor E64d, Chen et al. (6) find that cathepsin S plays a part in the signaling of TGF-β1, myofibroblast differentiation, and ECM creation and modulation in myocardial infarction (MI). The researchers describe how cathepsin S participates in regulating scar formation in the infarcted myocardium and preserve left ventricular function after experimentally induced MI. Further, Shi et al. (13) find that growth of new blood vessels is abnormal during wound repair in a cathepsin S-deficient mouse model, despite regular endothelial and fibroblast growth factor levels. Their results show that when cathepsin S activity is inhibited, the formation of microtubules is reduced, suggesting an essential role of cathepsin S in ECM degradation during vessel formation and repair.

Sasaki et al. (12) suggest that cathepsin S derived from macrophages are involved in the mechanisms that lead to the vulnerability of atherosclerotic plaque with increased levels of cathepsin S found in plaque. Knowing of angiotensin II as a player in vascular homeostasis, the researchers elucidate to angiotensin II type 1 receptor (AT1) blocker, olmesartan, as maintaining the stability of plaques, while simultaneously suppressing cathepsin S and macrophage activity. The authors then suggest the use of olmesartan as a treatment intervention in reducing cardiovascular consequences.

Cheng et al. (1) in a comprehensive review of the literature on the contributions of cathepsins in atherosclerosis-based vascular disease find strong evidence implicating cathepsins in related mechanisms of action. The researchers highlight that feasibility studies looking at cathepsins as diagnostic tools have shown promising results in the use of cathepsins S and L, and the endogenous inhibitor cystatin C as biomarkers for determining coronary artery disease and the formation of aneurysms.

In abdominal aortic aneurysm (AAA), often described for its wide degradation of the aortic wall matrix, remodeling and further rupturing of the wall, Qin et al. (9) in a murine model, provide evidence of cathepsin S’s role in the formation of AAA and suggest cathepsin S as a new therapeutic target for preventing AAAs in humans. Additionally, Lv et al. (10) test plasma samples of 476 male patients with AAA and 200 age-matched male controls as part of an ongoing randomized trial of more than 50,000 men aged 65–74 in Denmark. The researchers find in this population a correlation between plasma cathepsin S, cystatin C, aortic diameter, and the lowest ankle–brachial index. Their results suggest these parameters could be used as biomarkers for assessing arterial diseases and AAA, highlighting that more advanced cases of AAA would contain higher levels of cathepsin S in the AAA lesions as well as in circulation.

Important to highlight is that investigators suggest future interventions in calcification-prone individuals target inflammation and phosphate imbalance, together with the selective inhibition of cathepsin S as a treatment target (3, 11). Additionally, after review of evidence on cathepsins in atherosclerosis-based vascular disease, and feasibility studies of cathepsins as potential diagnostic tools, Cheng et al. (1) conclude that cathepsins S and L and endogenous inhibitor cystatin C could be used as biomarkers in determining the existence of coronary artery disease and the formation of aneurysms clinically.

### Cathepsin S Inhibition

Other researchers have also inquired about various cathepsin S inhibitors as treatment for cardiovascular disorders. Ahmad and Siddiqi (28) perform simulations of the molecule’s docking and dynamics to better understand the molecular mechanism of action of the cathepsin S inhibitor RO5444101. The researchers demonstrated a selectivity of this inhibitor for cathepsin S rather than cathepsin L1/L, cathepsin L2/V, and cathepsin K, claiming that the cathepsin S compound is more stable and involves more protein–molecule interactions. Figueiredo et al. (4) found that the systemic inhibition of cathepsin S by the compound RO5444101 attenuated the progression of atherosclerotic lesions in high-fat high-cholesterol fed apolipoprotein E-deficient nephrectomized mice. The authors conclude that the cathepsin S inhibitor accomplishes this through the simultaneous reduction in immunoreactivity of cathepsin S, elastin degradation, plaque size, macrophage accumulation, growth differentiation factor-15, and alkaline phosphatase activity. These results suggest a potential role of cathepsin S in the treatment of atherosclerosis in patients with CKD by possibly reducing the progression of atherosclerotic lesions.

In addition, Rupanagudi et al. (29), in an experimental mouse model of systemic lupus erythematosus and lupus nephritis, find that cathepsin S inhibition by RO5461111 shows therapeutic benefits. The inhibition of cathepsin S significantly reduced the excessive autoimmune response found in this disease presentation by blocking the assembly of major histocompatibility complex class II molecules in T and B cells. The investigators suggest that the inhibition of cathepsin S protects in the progression of lupus nephritis and could be useful in other autoimmune diseases.

Further, Payne et al. (30) assess the tolerability of safety of the cathepsin S inhibitor, LY3000328 in a phase 1, placebo-controlled study. The researchers use a single escalating dose ranging from 1 to 300 mg with 21 healthy male volunteers and find that the compound was quickly cleared from plasma within 12–13 h. They also found that the compound produced a transient decrease in plasma cathepsin S activity followed by a more prolonged increase in plasma cathepsin S mass. With this, the authors suggest that future studies include longer post-dose measurements to assess activity and impact. Additionally, Jadhav et al. (31), using an experimental CaCl2-induced AAA mouse model, report that cathepsin inhibitor LY3000328 binds to subsites without forming covalent interactions. The researchers report that among the tested compounds, LY3000328 was selected for clinical development, and may provide a new clinical treatment for AAA.

Hilpert et al. (32) further account the development of “Potent and Selective Cathepsin S Inhibitors Containing Different Central Cyclic Scaffolds.” In a transgenic mouse model of antigen presentation (DO10.11), the authors show a reduction in the production of the immune system cytokine interleukin (IL)-2 by one of the cathepsin S inhibitors studied through dose-dependent studies. Finally, Samokhin et al. (33) investigate the effects of a specific cathepsin S inhibitor in atherosclerotic plaque progression in an apoe–deficient mice fed a high-fat diet. The inhibition of cathepsin S showed protection of atherosclerotic activity. The researchers indicated that this protection is depicted in the decrease of atherosclerotic plaque size, the number of elastin lamina breaks, the numbers of plaque macrophages and buried fibrous caps. To highlight is that the researchers found a 36 and 68% reduction in plaque size, 60 and 75% in elastin breaks in males vs. females, respectively, and caphepsin S-deficient mice showed a decrease of close to 90%. These results further demonstrate the potential of cathepsin S inhibition as therapy.

The described research has looked at various cathepsin S inhibitors such as RO5444101, RO5461111, LY3000328, among others, to understand mechanism, evaluate clearance from plasma, test toxicity, and investigate whether these inhibitors may be good options as clinical treatment targets. Researchers have also demonstrated how the inhibition of cathepsin S decreased the size of atherosclerotic plaques showing cathepsin S’s potential role as a therapeutic option.

### Cathepsin S and Inflammation

In terms of the association between cathepsin S and inflammation, Jobs et al. (27) investigate in a community-based cohort of elderly men, whether there is an existing association between serum levels of cathepsin S and markers of inflammation mediated by cytokines. The authors then find that higher levels of cathepsin S were associated with higher C-reactive protein and higher serum IL-6 levels, both inflammatory markers. This association was persistent at a reassessment after 7 years from the initial baseline levels acquired. The researchers find that an interplay between cathepsin S and inflammation markers are present even in normal-weight individuals.

Further, in two independent cohorts of elderly men and women, Jobs et al. (26) sought to evaluate associations between circulating cathepsin S levels and mortality, considering that experimental studies have suggested the connection between cathepsin S activity and the development of CVD through the increase in the formation of and destabilization of atherosclerotic plaque. The researchers find that higher serum cathepsin S levels were associated with an increased mortality risk in a linear regression model. Further, in one of the independent cohorts, the researchers found that cathepsin S was independently associated with CVD and cancer, and suggest future studies should evaluate cathepsin S’s potential clinical utility.

Lafarge et al. (16), in a review of current literature, attempt to understand further the links between obesity, metabolic disease, and CVD. Knowing that adipose tissue produces a number of pro-inflammatory factors, the group find that the gene encoding cathepsin S is one of the most unregulated in the adipose tissue of obese subjects. Further, the researchers also add that cathepsin S is positively correlated with body mass index. To conclude, they highlight that future inquiries are needed to establish whether cathepsin inhibitors could be beneficial in reducing metabolic and cardiovascular comorbidities in the obese.

Additionally, Naour et al. (18) in a prospective study with two independent cohorts of obese females find that when looking at cathepsin S, L, and K, that obese subjects have a twofold increase in cathepsin S in adipose tissue, as compared to normal-weight control subjects, and an increased rate of cathepsin S release in adipose tissue. However, it remains unknown whether the inhibition of cathepsin S in obesity can reduce cardiovascular risk or improve the metabolic status of obese patients.

### Cathepsin S and Renal Disease

Aikawa et al. (3) demonstrate that CKD accelerates cathepsin S-induced atherosclerotic and aortic valve inflammation and calcification in apolipoprotein-deficient mice. On the connection between cathepsin S and renal disease, a study by Steubl et al. (35) finds that as glomerular filtration rates decline, cathepsin S and markers of inflammation-related endothelial dysfunction increase. This indicates that cathepsin S activity increases with CKD progression, suggesting that cathepsin S may be a therapeutic target to prevent cardiovascular complications in CKD. Huang et al. (34) reveal a role of cathepsin S in epidermal growth factor receptors (EGFR) signaling regulation. EGFR expression has been reported as increased in various tumors of the bladder, colon, ovarian, and kidney. Thus, the researchers argue for the clinical evaluation of cathepsin S and EGFR tyrosine kinase inhibitors in combination.

Further, Carlsson et al. (36) in a longitudinal cohort study of 207 patients undergoing hemodialysis found that cathepsin S and L were associated with receptors for tumor necrosis factors. The researchers conclude that the high levels of endostatin, cathepsins S and L, and their associations with tumor necrosis factors warrant further studies within this population exploring mortality, and pathways involved in end-stage renal disease. Additionally, Smith et al. (38) analyze in 200 patients with stages 3 and 4 CKD and a subgroup of 65 patients, elastin-derived peptides, their endogenous inhibitors, and aortic pulse wave velocity over a 36-month period. The researchers find that higher serum elastin-derived peptide levels were independently associated with increased all-cause mortality.

### Cathepsin S and Calcification

Cardiovascular calcification is described as a disease resulting from the disarray of an individual’s mineral metabolism where the buildup of minerals such as calcium and phosphate can lead to the vessel hardening and disruption of normal physiological processes (3). At the population level, a growing burden of epidemiologic factors such as aging, hypercholesterolemia, and renal insufficiency have led to an increased prevalence of arterial and aortic valve calcification.

Further, of important clinical concern has been the progression of calcification to even more debilitating conditions such as plaque rupture or aortic valve stenosis. Of additional concern is the fact that there are currently no treatment options available for reducing or avoiding these advanced conditions, beyond valve replacement (2).

In a study looking at the role of fibroblasts in medial vascular calcification, Simionescu et al. (43) find that calcified nodules are formed in the presence of elastin degradation products and TGF-β1. The researchers show that elastin degradation peptides can induce calcification of mesenchymal cells *in vitro*. These findings suggest that elastin degradation could in fact induce calcification of vascular SMCs and valvular myofibroblasts and thus mediate calcification.

A few other studies have also suggested that in the presence of mineral discrepancy, cathepsin S can participate in cardiovascular calcification where extracellular vesicles could then serve as loci for microcalcifications (44–49). The above described studies as well as studies by Aikawa et al. (3) and Figueiredo et al. (4) suggest the role of cathepsin S in cardiovascular calcification associated with mineral imbalance found in diabetes and CKD.

## Discussion

Multiple studies have indicated that cathepsin S activity increases with the progression of CKD and have highlighted cathepsin S inhibition as a therapeutic target in the prevention of cardiovascular complications for this patient population. Patients with CKD face a deleterious feedback loop composed and compounded by chronic inflammation and a consistent imbalance in calcium phosphate serum levels. This harmful combination accelerates and exacerbates the evolution of atherosclerosis in patients with CKD. In atherosclerotic lesions, cathepsin S, one of the most potent mammalian elastases, is highly expressed. Macrophages are then set in action as an essential part of the innate immune response, further releasing high levels of cathepsin S.

With the progression of kidney failure in patients with CKD, uremia and uremic toxins, produced during the deterioration of multiple biological functions could lead to the apoptosis and early damage of vessel wall. Extracellular vesicles could then serve as loci for microcalcifications (44–49). However, in the face of altered mineral metabolism, this could also lead to a set of pathological reactions including calcification of medial SMCs and elastin. Damaged kidneys and abnormal hormone levels in CKD cause calcium and phosphorus levels in the blood to be out of balance. This disruption and potential damage more commonly occurs in people with kidney failure receiving hemodialysis, who are more prone to aortic valve calcification and stenosis, needing valve replacement before undergoing kidney transplantation. This in itself could lead to increased cardiovascular mortality for those with CKD. Elevations in serum calcium and phosphate levels, such as what occurs with patients under dialysis with CKD, or as a result of calcium–phosphate binder use, or vitamin D treatment (11), can lead to devastating circumstances, and is even more critical for this patient population.

**Figure 1:**
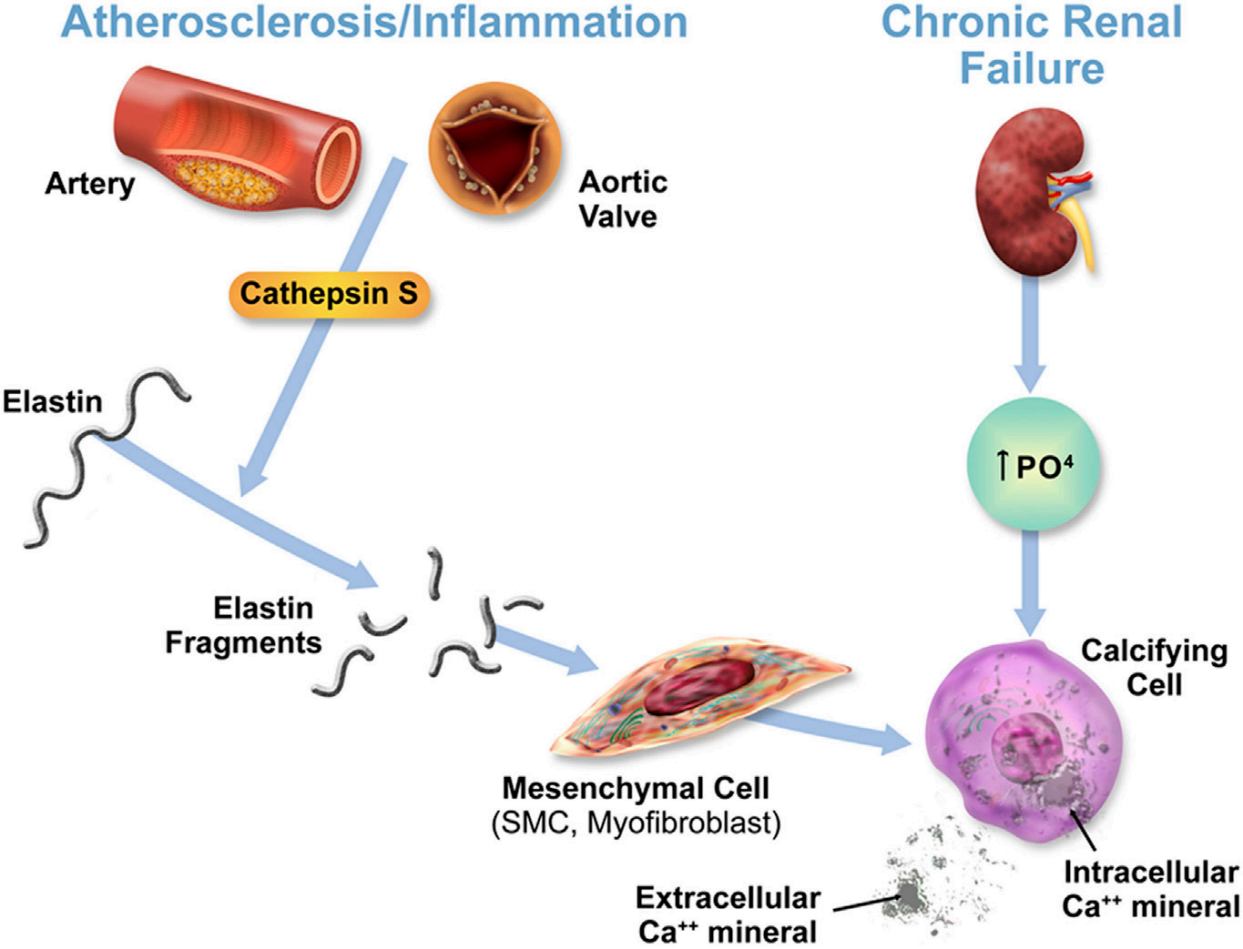
Acceleration of cardiovascular calcification through cathepsin S-associated elastin degradation in a CKD milieu. This figure is reproduced from Aikawa et al. (3), with the author’s permission.

Cathepsin S, through the cleavage of elastin and generation of bioactive elastin peptides, boosts cardiovascular inflammation and calcification. Changes in mineral imbalance, along with the elevation of cathepsin S, may lead to the transformation of mesenchymal cells, including vascular SMCs and interstitial cells to an osteochondrogenic, or stiffened phenotype, thus accelerating calcification even further (Figure 1) (3). Through its role in cardiovascular calcification in its association with calcium and phosphate imbalance, cathepsin S is thus involved in the feedback loop between these two biological processes predominant in CKD.

Therefore, as demonstrated in these investigations, cathepsin S may play a deleterious role and be the culprit for the initiation of calcification. This transition to a calcified state could be induced either *via* increased elastolytic activity and production of elastic peptides that directly induce smooth muscle cell or interstitial cell differentiation toward osteogenic bone-like phenotype, or *via* the induced release of extracellular vesicles or apoptotic bodies that could serve as loci resulting in dystrophic calcification (50).

Aortic valve calcification through the hardening of the valve reduces the movement of aortic valve leaflets, impacting and weakening cardiac function. Because mature aortic valves have an elastin-rich, multilayered structure and can develop inflammatory lesions that recapitulate features of atherosclerotic plaques, researchers have proposed that similar mechanisms of cathepsin S-associated elastin degradation contribute to the development of calcific aortic valve disease (3).

Facing this deleterious feedback loop compounded by chronic inflammation and a consistent imbalance in calcium–phosphate serum levels, patients with CKD are found in a harmful combination that would further accelerate and exacerbate the evolution of cardiovascular calcification (51, 52). Therefore, researchers have suggested that the early diagnosis and intervention toward interfering the progression of aortic valve calcification could provide immense clinical benefits.

The studies evaluated in this review have provided further evidence of the potential of the inhibition of cathepsin S as intervention toward reducing plaque development and diminishing the effects of CVD especially for patients with CKD.

## Author Contributions

BS: data analysis and manuscript writing; JF: data analysis; EA: critical review of the manuscript, supervision, and funding.

## Conflict of Interest Statement

The authors declare that the research was conducted in the absence of any commercial or financial relationships that could be construed as a potential conflict of interest.
